# Genetic redundancy of GATA factors in the extraembryonic trophoblast lineage ensures the progression of preimplantation and postimplantation mammalian development

**DOI:** 10.1242/dev.145318

**Published:** 2017-03-01

**Authors:** Pratik Home, Ram Parikshan Kumar, Avishek Ganguly, Biswarup Saha, Jessica Milano-Foster, Bhaswati Bhattacharya, Soma Ray, Sumedha Gunewardena, Arindam Paul, Sally A. Camper, Patrick E. Fields, Soumen Paul

**Affiliations:** 1 Department of Pathology and Laboratory Medicine andInstitute for Reproductive Health and Regenerative Medicine, University of Kansas Medical Center, Kansas City, KS 66160, USA; 2 Department of Developmental Neurobiology, St. Jude Children's Research Hospital, Memphis, TN 38105, USA; 3 North Texas Eye Research Institute, University of North Texas Health Science Center, Fort Worth, TX 76107, USA; 4 Department of Molecular and Integrative Physiology, Department of Biostatistics, University of Kansas Medical Center, Kansas City, KS 66160, USA; 5 Department of Human Genetics, University of Michigan, Ann Arbor, MI 48109, USA

**Keywords:** Mammalian development, GATA2, GATA3, Trophoblast stem cells, Placenta, Mouse, Human

## Abstract

GATA transcription factors are implicated in establishing cell fate during mammalian development. In early mammalian embryos, GATA3 is selectively expressed in the extraembryonic trophoblast lineage and regulates gene expression to promote trophoblast fate. However, trophoblast-specific GATA3 function is dispensable for early mammalian development. Here, using dual conditional knockout mice, we show that genetic redundancy of *Gata3* with paralog *Gata2* in trophoblast progenitors ensures the successful progression of both pre- and postimplantation mammalian development. Stage-specific gene deletion in trophoblasts reveals that loss of both GATA genes, but not either alone, leads to embryonic lethality prior to the onset of their expression within the embryo proper. Using ChIP-seq and RNA-seq analyses, we define the global targets of GATA2/GATA3 and show that they directly regulate a large number of common genes to orchestrate stem versus differentiated trophoblast fate. In trophoblast progenitors, GATA factors directly regulate BMP4, Nodal and Wnt signaling components that promote embryonic-extraembryonic signaling cross-talk, which is essential for the development of the embryo proper. Our study provides genetic evidence that impairment of trophoblast-specific GATA2/GATA3 function could lead to early pregnancy failure.

## Abstract (precis)

**Summary:** During trophoblast development in mice, GATA2 and GATA3 act synergistically by directly regulating a large number of common genes, and together are important to ensure trophoblast lineage progression.

## INTRODUCTION

The extraembryonic trophoblast cell lineage is unique to mammals and is essential for successful progression of mammalian reproduction. Trophoblast cells only exist during embryonic development and originate during the first cell fate decision in preimplantation embryos (Cockburn and Rossant, 2010; Pfeffer and Pearton, 2012; Roberts and Fisher, 2011; Rossant and Cross, 2001). Subsequently, trophoblast cells mediate implantation of the developing embryo into the uterus and establish a maternal-fetal interface for vascular connection with the mother for nutrient and gas transport to the embryo (Rossant and Cross, 2001). Failure in the determination of the trophoblast lineage during preimplantation development leads to defective embryo implantation (Cockburn and Rossant, 2010; Pfeffer and Pearton, 2012; Roberts and Fisher, 2011; Rossant and Cross, 2001), which is a leading cause of infertility. After implantation, defective development and function of trophoblast progenitors lead to either early pregnancy failure or pregnancy-associated complications such as intrauterine growth retardation (IUGR), pre-eclampsia (Myatt, 2006; Pfeffer and Pearton, 2012; Redman and Sargent, 2005; Rossant and Cross, 2001), or cause postnatal or adult diseases (Gluckman et al., 2008).

Development of the trophoblast cell lineage is a multistep process (Fig. S1) and begins with the establishment of the trophectoderm (TE) in blastocysts. The TE mediates blastocyst implantation and is the source of trophoblast stem and progenitor cells (TSPCs). In the early postimplantation mouse embryo, TSPCs proliferate and differentiate to develop the extraembryonic ectoderm (ExE). Later, at about embryonic day (E) 7.0-8.0, the ectoplacental cone (EPC) and chorion develop. Subsequently, lineage-specific trophoblast progenitors arise from TSPCs, which differentiate to specialized trophoblast subtypes leading to successful placentation. Thus, trophoblast lineage development relies upon the proper spatial and temporal regulation of gene expression during: (1) TE development in the preimplantation embryo; (2) maintenance of self-renewal within TSPCs of the early postimplantation embryo; and (3) subsequent differentiation of trophoblast progenitors to specialized trophoblast subtypes of the mature placenta.

Studies with gene knockout mice and mouse trophoblast stem cells (TSCs) implicated several transcription factors, including GATA3, in the regulation of trophoblast lineage development (Barak et al., 1999; Hemberger et al., 2010; Home et al., 2009; Keramari et al., 2010; Nishioka et al., 2008; Ralston and Rossant, 2008; Russ et al., 2000; Strumpf, 2005; Yagi et al., 2007). Our and other laboratories have reported that GATA3 is selectively expressed in extraembryonic TE and TSPCs during early mouse development and is involved in TE-specific gene regulation (Home et al., 2009; Ralston et al., 2010). Also, ectopic expression of *Gata3* in mouse embryonic stem cells (ESCs) or mouse fibroblasts is able to instigate trophoblast fate (Benchetrit et al., 2015; Kubaczka et al., 2015; Ralston et al., 2010). However, *Gata3-*null mouse embryos die at ∼E11.5 due to defective neuroendocrine system development (Lim et al., 2000; Pandolfi et al., 1995), indicating that trophoblast-specific GATA3 function is not essential for early mammalian development.

Like GATA3, GATA2 is also implicated in the regulation of a few trophoblast genes in the mouse placenta (Bai et al., 2011; Ma et al., 1997; Ray et al., 2009). Both GATA2 and GATA3 are selectively expressed in the TE of the preimplantation human embryo (Assou et al., 2012; Blakeley et al., 2015). However, *Gata2-*null mouse embryos die at ∼E10.5 due to defective hematopoiesis (Tsai and Orkin, 1997), indicating that, like GATA3, trophoblast-specific GATA2 function is not essential for early mammalian development. Thus, although both GATA2 and GATA3 are implicated in gene regulation at different stages of trophoblast lineage development, individual functions of GATA2 or GATA3 are dispensable for this process.

As GATA factors often show functional redundancy during the development of other tissues (Fujiwara et al., 2004; Peterkin et al., 2007), we hypothesized that GATA2 and GATA3 might exhibit functional redundancy in the developing trophoblast lineage. To test this hypothesis, we established inducible gene knockout mice, in which *Gata2* and *Gata3* could be conditionally deleted individually or in combination. We discovered that combinatorial functions of GATA2 and GATA3 are important to establish trophoblast lineage development in both pre- and postimplantation embryos. Both GATA2 and GATA3 target transcriptionally active and silent genes to orchestrate developmental stage-specific gene expression programs in TSPCs, which in turn ensure both pre- and early postimplantation mammalian development. Owing to the lack of an early trophoblast phenotype in these gene knockouts, it remains unknown whether trophoblast-specific functions of GATA2 and GATA3 are essential to assure the early development of mammalian embryos.

## RESULTS

### Expression of GATA2 and GATA3 is restricted to extraembryonic trophoblast cells during early mouse development

**Fig. 1.**
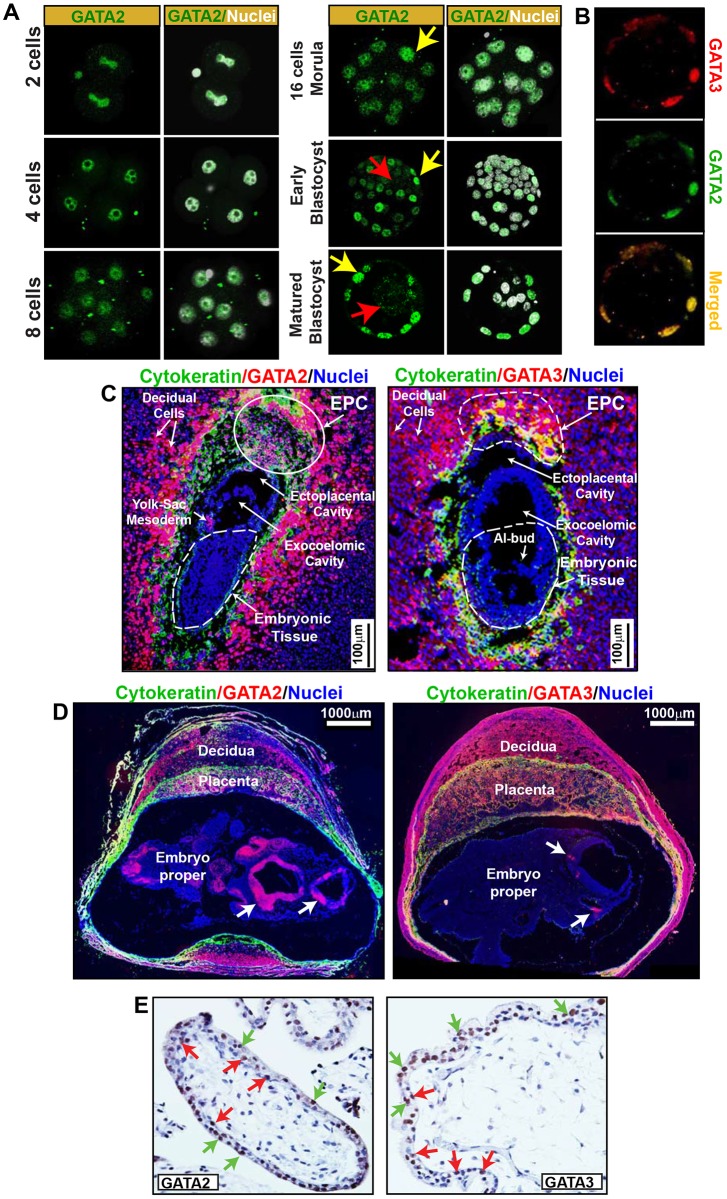
**GATA2 and GATA3 are selectively expressed in trophoblast cells of preimplantation and early postimplantation mammalian embryos.** (A) Immunofluorescence images showing GATA2 expression at different stages of preimplantation mouse development. During blastulation, GATA2 is induced in the outer TE lineage (yellow arrows) but is repressed in the inner cell mass lineage (red arrows). (B) A mouse blastocyst showing that both GATA2 and GATA3 are selectively expressed within the TE lineage. (C) ∼E7.5 mouse implantation sites showing pan-cytokeratin, GATA2 (left), GATA3 (right) and nuclei (DAPI). Outlined areas show ectoplacental cone (EPC) and embryonic tissue. (D) E10.5 mouse implantation sites showing pan-cytokeratin, GATA2 (left), GATA3 (right) and nuclei (DAPI). At this stage, GATA2 and GATA3 are expressed in both embryonic cells (arrows) and extraembryonic tissues. (E) Immunohistochemistry showing that both GATA2 (left) and GATA3 (right) are selectively expressed within trophoblast cells [both cytotrophoblast progenitors (red arrows) and syncytiotrophoblasts (green arrows)] of a first-trimester (8 week) human placenta.

During preimplantation mouse development, *Gata3* mRNA expression is induced at the 4-cell stage, and GATA3 protein is detectable during the 8- to 16-cell transition (Home et al., 2009; Ralston et al., 2010). However, in a mature blastocyst, GATA3 mRNA and protein expression becomes restricted to the TE lineage (Home et al., 2009; Ralston et al., 2010). Recently, other studies showed that both *GATA2* and *GATA3* mRNAs are selectively expressed within the TE lineage of the human preimplantation embryo (Assou et al., 2012; Blakeley et al., 2015). However, GATA2 protein expression is not well documented during preimplantation development. We therefore followed GATA2 protein expression at different stages of mouse preimplantation development. We found low levels of GATA2 protein in blastomeres of 2- to 16-cell embryos. However, GATA2 expression was upregulated in outer TE lineage cells and repressed in the inner cell mass during blastocyst maturation (Fig. 1A). In mature blastocysts, both GATA2 and GATA3 were only expressed within the TE lineage (Fig. 1B).

We also tested GATA2 and GATA3 protein expression in early postimplantation mouse embryos. Up to Theiler stage 10c (∼E7.25), expression of GATA2 and GATA3 was mostly confined to the extraembryonic trophoblast cells, including TSPCs within the EPC (Fig. 1C). At ∼E7.25-7.5, a few cells of the extraembryonic yolk sac mesoderm also began to express GATA2 protein (Fig. 1C). However, GATA2 and GATA3 proteins were not expressed in the embryonic cells prior to E7.5. Subsequently, GATA2 and GATA3 expression was induced in the embryo proper and also maintained in trophoblast cells (Fig. 1D).

Thus, our study confirmed a trophoblast-specific expression pattern of GATA2 and GATA3 during blastocyst maturation and early postimplantation development in the mouse. We also examined GATA2 and GATA3 expression within trophoblast progenitors of developing first-trimester human placenta and found that the simultaneous expression of GATA2 and GATA3 in cytotrophoblast progenitors is a conserved event during early human development (Fig. 1E).

### GATA factors are essential to establish a functional TE lineage during preimplantation mouse development

To test the functional importance of GATA2 and GATA3 during early mouse development, we studied conditional knockout mice in which *Gata2* and *Gata3* could be efficiently deleted individually (*Gata2*-KO or *Gata3*-KO) or in combination (*Gata*-DKO), by inducing the activity of a Cre-ERT2 recombinant protein with tamoxifen (Fig. S2). Given that the expression of both GATA factors is restricted to within the developing trophoblast lineage of the early mouse embryo, this inducible gene knockout system allowed us to study trophoblast-specific GATA2/GATA3 functions at distinct stages of early mouse development.

**Fig. 2.**
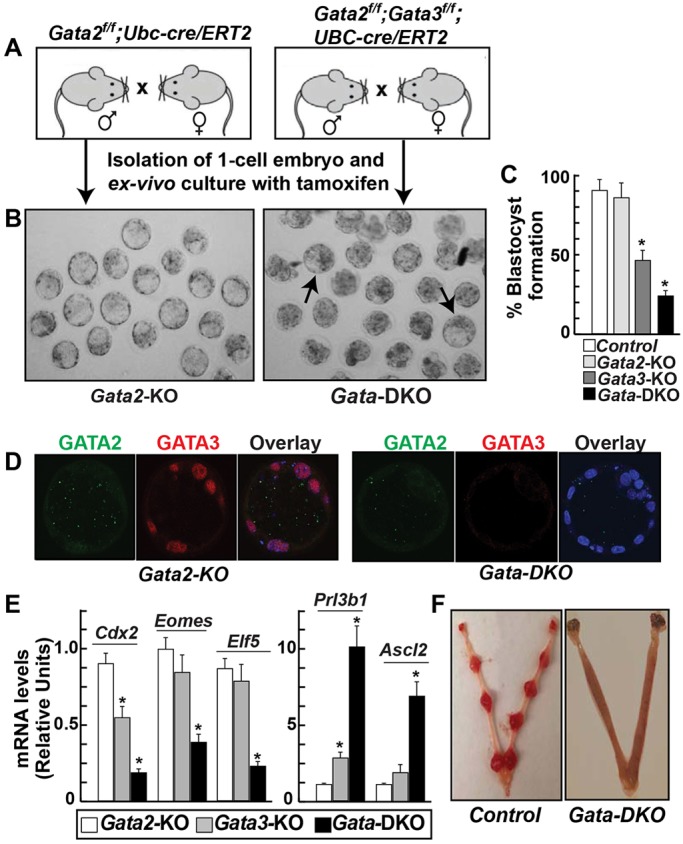
**Combinatorial loss of GATA2 and GATA3 impairs functional TE development.** (A) Experimental strategy to define the importance of GATA factors during mouse preimplantation development. (B) Micrographs show that the loss of GATA2 is dispensable for blastocyst maturation, whereas loss of both GATA2 and GATA3 results in a partial defect in blastocyst formation. Arrows indicate matured blastocysts with deleted GATA genes. (C) The percentage of preimplantation embryos that matured to the blastocyst stage upon loss of GATA factors. Mean±s.e., *n*=3, *P≤0.01. (D) Immunofluorescence confirmed the loss of GATA2 expression in *Gata2*-KO blastocysts and loss of both GATA2 and GATA3 expression in *Gata*-DKO blastocysts. Blue stain is DAPI. (E) Analysis of mRNA expression, showing significant changes in TE-specific genes in *Gata-*DKO embryos compared with *Gata2*-KO or *Gata3*-KO embryos. The expression level of a gene in control embryos was considered 1. Mean±s.e., *n*=3, *P≤0.001. (F) Uterine horns from E7.5 pseudopregnant female mice that received control (left) or *Gata-*DKO (right) blastocysts. *Gata-*DKO blastocysts showed implantation failure.

Previously, using an RNAi strategy, we showed that GATA3 depletion in preimplantation mouse embryos partially impairs blastocyst maturation (Home et al., 2009). However, preimplantation mouse development in the absence of both GATA2 and GATA3 was not tested. Therefore, we began our study by examining the importance of individual as well as combinatorial GATA2/3 function during preimplantation mouse development. We isolated fertilized embryos at E0.5, induced GATA gene deletion with tamoxifen and monitored preimplantation development *ex vivo* (Fig. 2A-D). We found that GATA2 is dispensable for blastocyst maturation (Fig. 2B,C) and, similar to the RNAi findings, conditional deletion of *Gata3* partially affected blastocyst maturation (Fig. S3A). Interestingly, combinatorial loss of both GATA factors also resulted in a mixed preimplantation phenotype. A large number of *Gata*-DKO embryos failed to form blastocysts. However, several of the *Gata*-DKO embryos matured to the blastocyst stage (Fig. 2B,C) despite the fact that Cre-mediated gene excision resulted in the loss of both GATA proteins in those embryos (Fig. 2D).

Next, we tested whether *Gata*-DKO blastocysts have altered expression of TE-specific genes. Our analysis confirmed that the mRNA expression of several TE-specific genes, including the GATA targets *Cdx2*, *Eomes* and *Elf5*, was strongly downregulated (Fig. 2E) in *Gata*-DKO preimplantation embryos. By contrast, mRNA expression of *Prl3b1* and *Ascl2*, which are predominantly expressed in differentiated trophoblast cells, was highly induced in *Gata*-DKO preimplantation embryos (Fig. 2E). Interestingly, except for *Cdx2* mRNA expression in *Gata3-*KO embryos, none of these genes was significantly altered in expression in *Gata2-*KO or *Gata3-*KO embryos (Fig. 2E). *Cdx2* expression was repressed by ∼40% in *Gata3-*KO embryos and reduced by >80% in *Gata-*DKO embryos. These results indicated that although a few *Gata*-DKO preimplantation embryos could mature to the blastocyst stage, TE-specific gene expression is altered in those embryos. We next tested the *in utero* implantation efficiency of *Gata*-DKO blastocysts.

As continuous tamoxifen exposure could negatively affect the implantation efficiency of a blastocyst (Dao et al., 1996), we used two different experimental strategies to test implantation efficiency of *Gata*-DKO blastocysts. First, we ectopically expressed Cre recombinase in *Gata2^f/f^;Gata3^f/f^* preimplantation embryos via lentiviral transduction (Fig. S3B). We found that ectopic Cre-mediated excision of GATA genes also resulted in a mixed phenotype and several *Gata*-DKO embryos matured to the blastocyst stage (Fig. S3B). However, those *Gata*-DKO blastocysts failed to implant when they were transferred to the uterine horns of pseudopregnant surrogate female mice (Fig. 2F).

In the second approach, we transiently cultured both wild-type and *Gata2^f/f^;Gata3^f/f^;UBC-cre/ERT2* preimplantation embryos with tamoxifen (Fig. S4). The transient tamoxifen exposure ensured GATA gene deletion and defective blastocyst maturation in the majority of the *Gata*-DKO embryos (data not shown). We transferred transiently tamoxifen-exposed *Gata2^f/f^;Gata3^f/f^;UBC-cre/ERT2* and wild-type embryos, which matured to the blastocyst stage, to the uterine horns of pseudopregnant mice. Wild-type blastocysts with tamoxifen exposure readily implanted (Fig. S4), indicating that transient exposure to tamoxifen does not affect blastocyst implantation efficiency. However, blastocysts that developed from *Gata*-DKO blastocysts after transient exposure to tamoxifen failed to implant (Fig. S4). Collectively, these results indicated that although GATA2 and GATA3 functions are not essential for blastocoel cavitation they are required to maintain proper gene expression balance and implantation efficiency within the developing TE lineage.

### GATA2/GATA3 functions in the trophoblast lineage are essential for postimplantation mammalian development

As GATA2 and GATA3 are selectively expressed in TSPCs of the early postimplantation mouse embryo (Fig. 1), we also tested the importance of TSPC-specific GATA2/GATA3 function during early postimplantation development. For this study, we started tamoxifen treatment at ∼E5.5, as the presence of tamoxifen on or before E4.5 affects the implantation process (Bloxham and Pugh, 1977; Dao et al., 1996; Pugh and Sumano, 1982). Also, we crossed *Gata2^f/f^;Gata3^f/f^;UBC-cre/ERT2* males with *Gata2^f/f^;Gata3^f/f^* females to confine GATA gene deletion to within developing embryos.

**Fig. 3.**
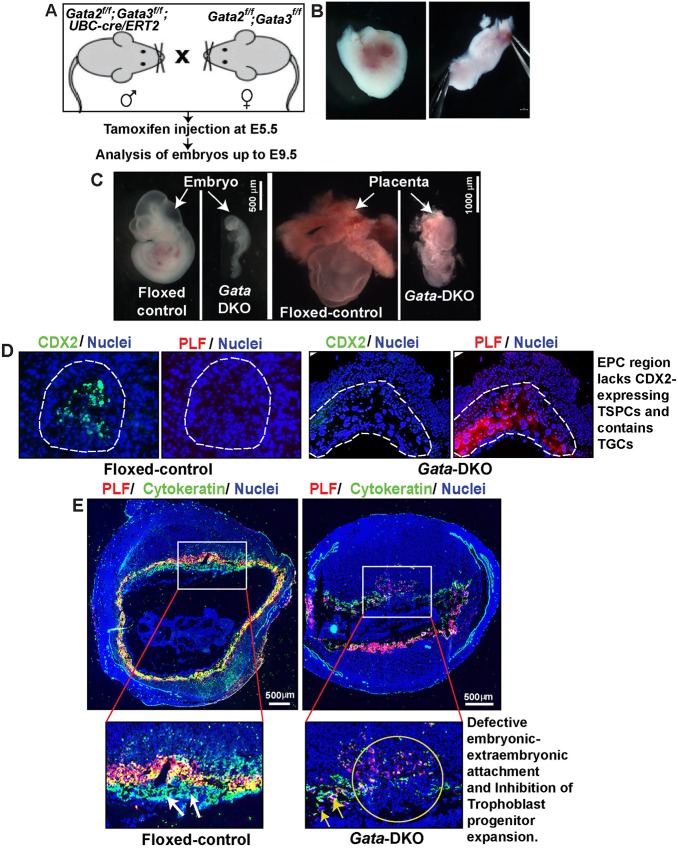
**Concurrent loss of GATA2 and GATA3 impairs early postimplantation development.** (A) Mating strategy to define the importance of GATA2 and GATA3 during early postimplantation mouse development. (B) An E7.5 *Gata*-DKO conceptus without the developing embryo inside. Left, before dissection; right, after dissection. (C) Control and *Gata*-DKO conceptuses were isolated at ∼E9.5 and examined for embryonic morphology (left) and placentation (right). The image of the *Gata*-DKO embryo is representative of a few embryos that developed to Theiler stage 12a. None of the *Gata*-DKO embryos developed beyond this stage. (D) Fluorescence images showing loss of CDX2-expressing (green) TSPCs but the presence of proliferin-expressing (PLF, also known as PRL2C2; red) TGCs within the prospective EPC region of an E7.5 *Gata*-DKO conceptus. (E) Placentation at control and *Gata-*DKO implantation sites was analyzed at ∼E9.5 (images are not on the same scale). Sections were immunostained with pan-cytokeratin (green) and for the TGC marker PLF (red). The maternal-fetal interface in the *Gata*-DKO embryo lacks trophoblast progenitors (insets, white arrows in control) but contains the primary TGC layer (yellow arrows). Also, unlike the control, the developmentally arrested *Gata*-DKO embryo proper is attached to the placentation site.

Individual deletion of *Gata2* or *Gata3* induces mouse embryonic lethality after E10.5 (Pandolfi et al., 1995; Tsai et al., 1994). Therefore, after inducing *Gata2*/*Gata3* deletion at E5.5, we monitored embryonic development on or before E9.5 (Fig. 3A). As expected, individual loss of GATA2 or GATA3 did not induce embryonic lethality by E9.5 (Table 1). However, combinatorial deletion of *Gata2* and *Gata3* at E5.5 prevented development of most of the embryos, resulting in embryonic death/loss at implantation sites before E7.5 (Fig. 3B). Although a few embryos developed, they died at ∼E7.5-8.0 and none developed beyond Theiler stage 12a (∼E8) (Fig. 3C). Furthermore, analysis of surviving *Gata*-DKO conceptuses revealed impaired placentation (Fig. 3C-E). ExE/EPC regions were not properly developed in *Gata*-DKO conceptuses and were characterized by near complete loss of CDX2-expressing TSPCs (Fig. 3D). Similarly, when analyzed at E9.5, the *Gata*-DKO conceptuses revealed defective embryonic-extraembryonic attachment and were characterized by near complete loss of trophoblast progenitors (Fig. 3E) at the maternal-fetal interface.

**Table 1.**
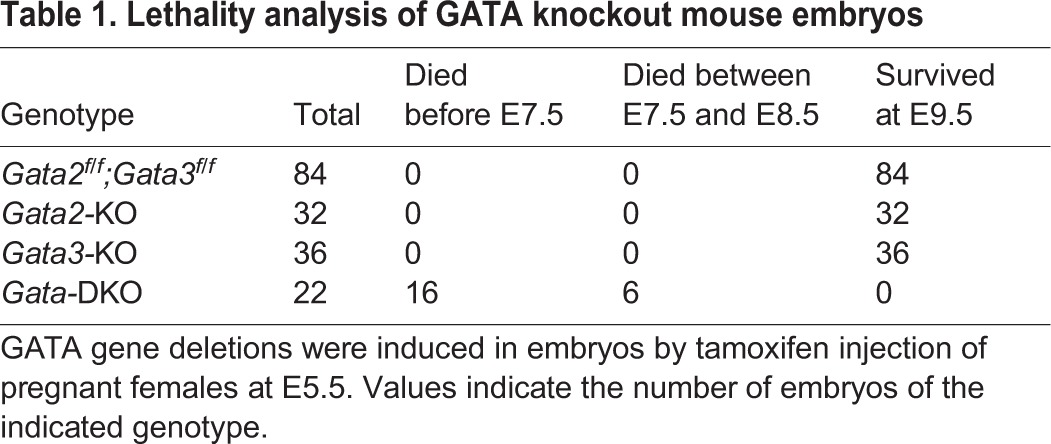
**Lethality analysis of GATA knockout mouse embryos**

**Fig. 4.**
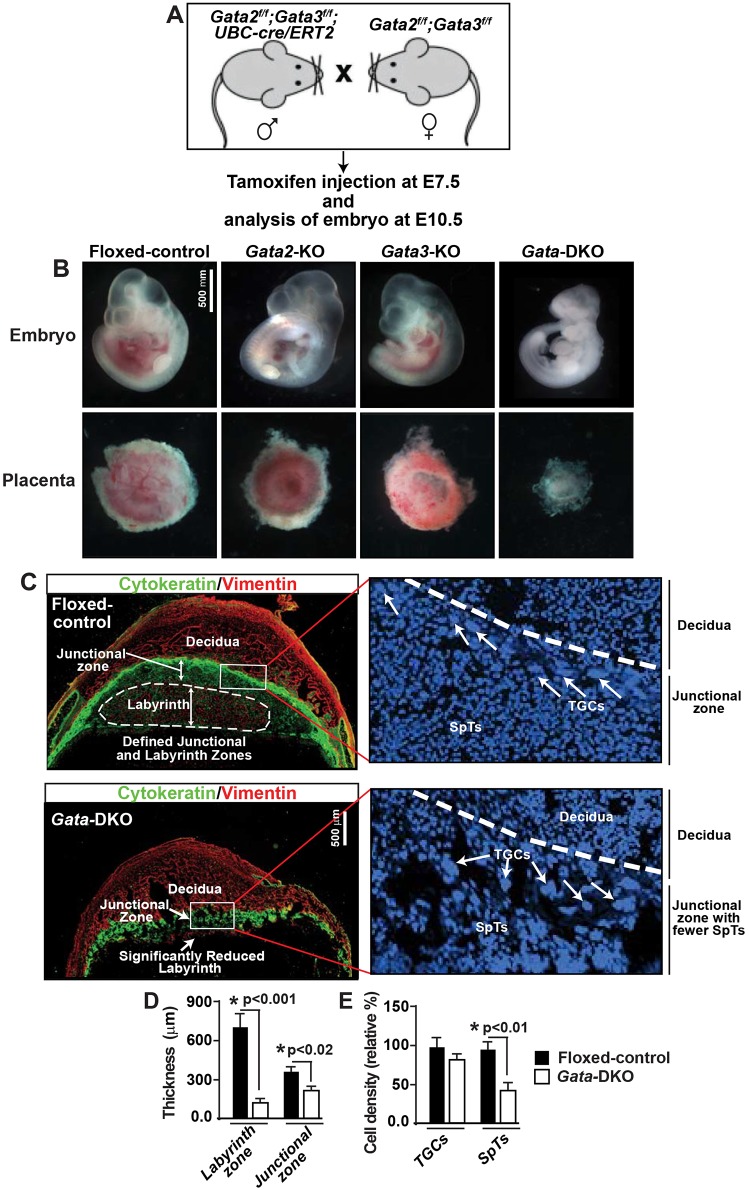
***Gata2* and *Gata3* ablations in differentiating trophoblast cells impair placental development and induce early embryonic lethality.** (A) Experimental strategy to define the importance of GATA factors during the differentiation of trophoblast progenitors to specialized trophoblast cells. (B) E10.5 embryos and placentae showing severe developmental defects in *Gata-*DKO compared with *Gata2*-KO, *Gata3*-KO and control embryos. (C) Immunofluorescence analyses showing a severe reduction of the labyrinth zone in the *Gata-*DKO placenta compared with the control. Pan-cytokeratin was used to mark the trophoblast layers, while vimentin was used to differentiate the junctional zone from the labyrinth zone and the uterine tissue. Insets show magnified regions of junctional zones with the presence of TGCs (arrows). (D) Quantitative analysis of the width of the labyrinth and junctional zones in control and *Gata*-DKO placentae. (E) Quantitative cell density analysis showing that the junctional zone in *Gata-*DKO placentae contains a similar number of TGCs to the control but is associated with significantly fewer spongiotrophoblast (SpT) cells. Error bars indicate mean±s.e., *n*=3.

Next, we asked whether combinatorial functions of GATA2 and GATA3 are essential for the development of differentiated trophoblast subtypes. In a developing mouse embryo, progenitors for differentiated trophoblast subtypes arise within the EPC and the chorionic ectoderm at ∼E8.0-8.5 (Fig. S1). Therefore, to test the importance of GATA factors during trophoblast progenitor differentiation, we induced *Gata2/Gata3* deletion at E7.5. At E7.5, GATA3 is not expressed in embryonic cells, and we were unable to determine GATA2 protein expression in the embryo proper before E7.5. Individual knockout of *Gata2* or *Gata3* induces mouse embryonic death on or after E10.5, so we analyzed embryonic development on or before E10.5 (Fig. 4A). Deletion of both GATA factors at E7.5 induced embryonic death at an earlier stage (∼E9.5) than the individual knockouts (Fig. 4B). Placenta development was not overtly affected in either *Gata2*-KO or *Gata3*-KO embryos (Fig. 4B). By contrast, placentae in *Gata*-DKO embryos were significantly smaller, with severely reduced labyrinth zones and significantly smaller junctional zones (Fig. 4C,D). Furthermore, junctional zones of *Gata*-DKO placentae were characterized by significant reduction of spongiotrophoblast (SpT) cells (Fig. 4E) without any significant loss in the trophoblast giant cell (TGC) population. Interestingly, complete loss of blood development (a more severe phenotype than that shown by *Gata2*-KO embryos) was also observed in *Gata*-DKO embryos and placentae (Fig. 4B). This complete loss of hematopoiesis is being studied in detail and will be reported elsewhere.

Collectively, conditional gene deletions at distinct developmental stages revealed that GATA2 and GATA3 functions in the extraembryonic trophoblast lineage are essential for both pre- and postimplantation embryonic development.

### GATA factors fine-tune gene expression to maintain trophoblast stem state

**Fig. 5.**
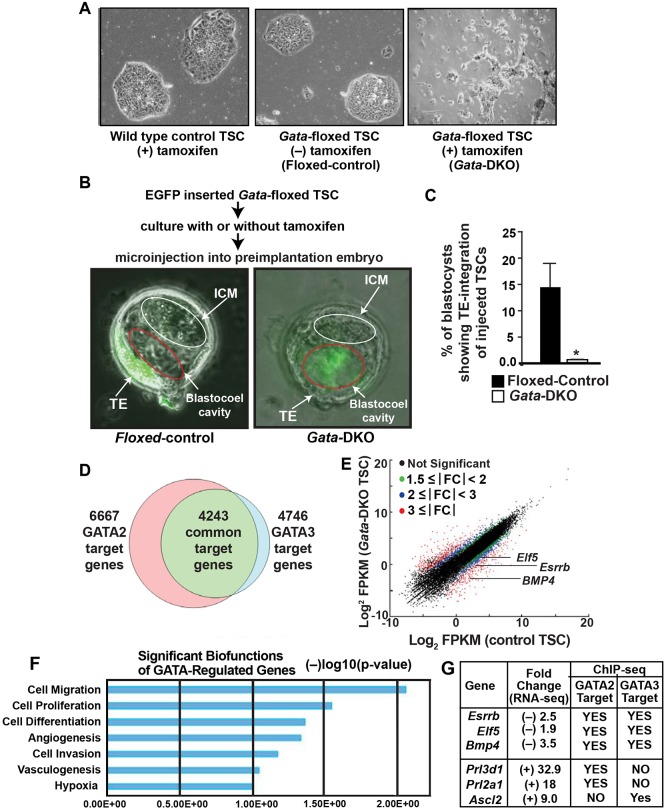
**Loss of GATA factors impairs the stem-state gene expression program in TSCs and primary TSPCs.** (A) Micrographs of control and *Gata2^f/f^;Gata3^f/f^;UBC-cre/ERT2* (*Gata*-floxed) TSC colonies in standard TSC culture conditions. Unlike wild-type TSCs (left), *Gata*-floxed TSCs that were cultured with tamoxifen to delete GATA genes (*Gata*-DKO, right) lost the stem-state colony morphology. *Gata*-floxed TSCs that were cultured without tamoxifen (floxed-control, middle) maintained stem-state colony morphology. (B) TE chimerism analyses of TSCs. Micrographs show that, unlike floxed-control TSCs, *Gata-*DKO TSCs failed to integrate into the TE and remained within the blastocoel cavity. ICM, inner cell mass. (C) Quantitative plot of TE chimerism analyses. Mean±s.e., *n*=3, *P≤0.001. (D) Venn diagram showing the number of genes that are direct targets of GATA2 and/or GATA3 and that also showed significant changes in mRNA expression in *Gata*-DKO TSCs. (E) The scatter plot shows fold change in mRNA expression of common GATA2 and GATA3 target genes, including the TSC-specific genes *Bmp4*, *Esrrb* and *Elf5*, in control versus *Gata*-DKO TSCs. (F) Ingenuity pathway analysis showing major biological functions associated with GATA2/GATA3-regulated genes in TSCs. (G) Downregulation of TSC-specific genes and upregulation of differentiated trophoblast markers in *Gata*-DKO TSCs. These representative genes are also direct targets of GATA2 and/or GATA3.

Trophoblast genes that are directly regulated by GATA2 and/or GATA3 are incompletely defined. An earlier study (Kidder and Palmer, 2010) used chromatin immunoprecipitation (ChIP) with DNA microarray hybridization analysis to investigate GATA3 binding at 28,000 promoter regions in mouse TSCs. However, global targets of GATA2, as well as GATA3 targets beyond the gene promoters, have not been defined in TSCs. Also, how GATA2 and GATA3 orchestrate different stages of trophoblast development is not well characterized. We hypothesized that, being pioneer transcription factors (Chen and Dent, 2014; Zaret and Carroll, 2011), GATA2 and GATA3 could target both open and silent chromatin regions in trophoblast cells to instigate developmental stage-specific gene expression programs, thereby establishing stem/progenitor versus differentiated cell fate. To test this hypothesis, we established TSCs in which *Gata2* and *Gata3* could be conditionally deleted individually (*Gata2-*KO and *Gata3*-KO) or in combination (*Gata*-DKO) (Fig. 5A, Fig. S5A,B) and asked whether a GATA factor-dependent transcriptional program is important to balance TSC self-renewal with differentiation. Our *in vitro* cell culture studies of TSCs maintained in undifferentiated culture with fibroblast growth factor 4 (FGF4) and heparin showed that loss of GATA2 and GATA3 induced TSC differentiation, leading to loss of stem-state colony morphology (Fig. 5A). Furthermore, the *Gata*-DKO TSCs failed to form chimera with the developing TE lineage when they were injected into developing preimplantation mouse embryos (Fig. 5B,C). By contrast, *Gata2-*KO TSCs or *Gata3-*KO TSCs maintained their self-renewal ability, although they showed a higher propensity for spontaneous differentiation than the wild-type control TSCs in standard TSC culture conditions (Fig. S5A). These studies indicated that whereas either GATA2 or GATA3 is dispensable, TSCs that have lost both GATA factors are unable to maintain the stem state.

To validate that GATA factors directly regulate key trophoblast genes we performed ChIP-seq analysis in wild-type control TSCs. We identified 12,949 GATA2 binding and 5638 GATA3 binding regions in the mouse TSC genome (Table S1A,B). RNA-seq analysis in control versus *Gata*-DKO TSCs showed that loss of both GATA factors altered the expression of 9775 genes by ≥1.5 fold (Table S2). A comparative analysis of the ChIP-seq and RNA-seq data revealed that, among those 9775 genes, ∼68% are direct targets of either GATA2 (6667 genes) or GATA3 (4746 genes) and that ∼43% (4243 genes) have both GATA2 and GATA3 occupancy at their chromatin domains (Table S3, Fig. 5D). Thus, our global genomics analysis revealed that ∼90% of GATA3 target genes (4243 out of 4746 genes) are also targets of GATA2 in TSCs, strongly supporting functional redundancy of these two GATA factors in gene regulation during early trophoblast development.

Our analyses showed altered expression of a large number of genes that are targeted by both GATA2 and GATA3 in TSCs (Fig. 5E) and revealed multi-modal biological functions of dual GATA-regulated genes (Fig. 5F). Several of those GATA target genes are implicated in trophoblast and placenta development. For example, mRNA expression of *Elf5*, *Esrrb* and *Bmp4*, which are direct targets of both GATA2 and GATA3 (Fig. 5G) and are implicated in TSC self-renewal, was strongly repressed in *Gata*-DKO TSCs (Fig. 5E). Our qRT-PCR analyses also validated the RNA-seq data (Fig. S5C). By contrast, mRNA expression of the GATA targets *Prl3d1* and *Prl2a1*, which are only expressed in terminally differentiated TGCs, and *Ascl2*, which is induced in SpT cells, was upregulated in *Gata*-DKO TSCs (Fig. 5G). Furthermore, ChIP-seq analyses confirmed that all these genes are direct targets of either GATA2 or GATA3 in TSCs (Fig. 5G). Thus, our ChIP-seq and RNA-seq analyses indicated that GATA2 and GATA3 mediate two important functions in undifferentiated TSCs: (1) to maintain the transcription of key genes that promote the trophoblast stem state; and (2) to suppress the transcription of genes that promote TSC differentiation.

**Fig. 6.**
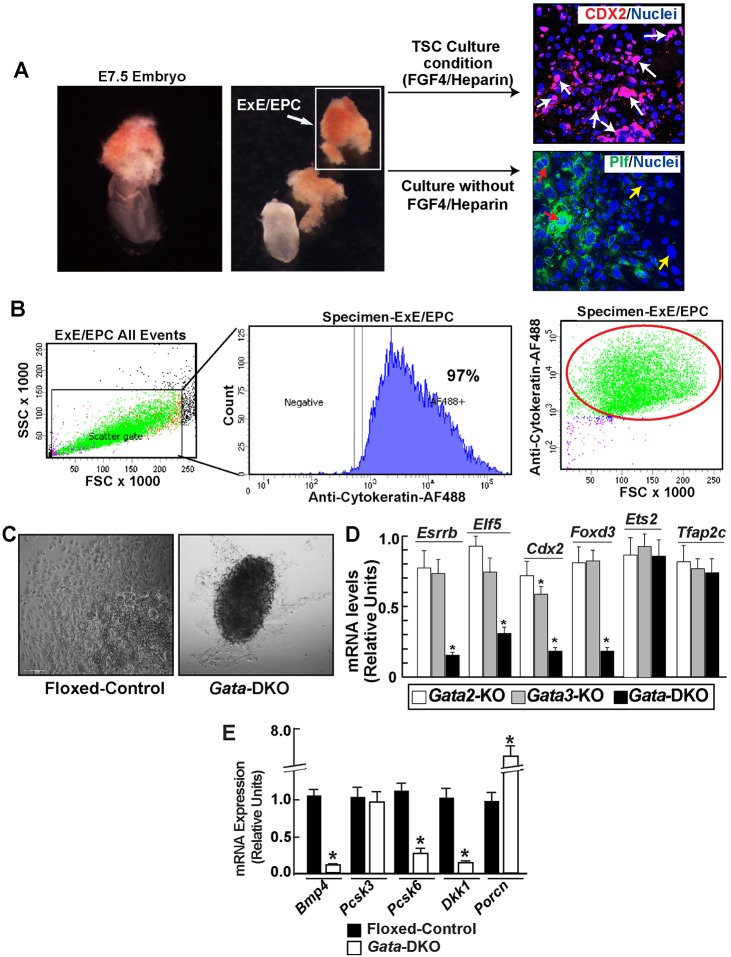
**GATA factors are required to activate developmental stage-specific gene expression in trophoblast progenitors to ensure differentiation and embryonic-extraembryonic cross-talk.** (A) ExE/EPC explant cultures, maintained in the presence of FGF4 and heparin, showed the presence of CDX2-expressing TSPCs (white arrows). Explants cultured in the absence of FGF4 undergo differentiation. In this condition, TSPCs differentiate into multiple cell types, including TGCs with (red arrows) or without (yellow arrows) expression of proliferin, a marker of parietal TGCs. (B) FACS using intracellular cytokeratin labeling shows that ExE/EPC explant culture consists of a high percentage of trophoblast cells. (C) Micrographs show *ex vivo* primary TSPC culture from floxed-control and *Gata*-DKO embryos treated with tamoxifen in the absence of FGF4. The *Gata-*DKO samples often showed reduced cell numbers, indicating abnormal proliferation of TSPCs. (D) Analysis of mRNA expression, showing significant reduction of several TSPC-specific genes in the *Gata-*DKO TSPCs compared with *Gata2*-KO and *Gata3*-KO. The expression level of a gene in control TSPCs was considered as 1. Mean±s.e., *n*=3, *P≤0.001. (E) Analysis of mRNA expression in control and *Gata-*DKO TSPCs showing altered expression of BMP4, Nodal and Wnt signaling genes that are implicated in successful gastrulation. Mean±s.e., *n*=3, *P≤0.01.

To confirm GATA-mediated regulation of stem-state genes, we studied gene expression in primary TSPCs. We established *ex vivo* explant cultures with ExE/EPC from early postimplantation mouse embryos (Fig. 6A). These explant cultures contain nearly pure (≥97%) primary trophoblast cells (Fig. 6B) and could be maintained in stem/progenitor states in the presence of FGF4 and heparin in TSC culture conditions (Fig. 6A). Also, in the absence of FGF4 and heparin, TSPCs in the explant culture undergo differentiation (Fig. 6A).

We performed gene expression analysis with these primary TSPCs after maintaining them in TSC culture conditions. We found that loss of both GATA factors often impairs the expansion of primary TSPCs (Fig. 6C) and represses mRNA expression of several TSC/TSPC-specific genes, including *Esrrb*, *Elf5* and *Cdx2* (Fig. 6D). Also, the expression of *Foxd3* (a GATA target), which is important for TSPC self-renewal (Tompers et al., 2005), was strongly downregulated in *Gata-*DKO TSPCs. By contrast, the expression of these genes was either unaltered or only marginally affected in *Gata2-*KO or *Gata3-*KO TSPCs (Fig. 6D). Interestingly, mRNA expression of *Ets2* (a GATA target) and *Tfap2c*, which are implicated in the maintenance of TSPCs in the early postimplantation embryo (Choi et al., 2012; Georgiades and Rossant, 2006; Kuckenberg et al., 2012), was not significantly altered in *Gata*-DKO TSPCs (Fig. 6D).

Collectively, our studies in *Gata*-DKO TSCs and primary TSPCs strongly indicated that functional redundancy of GATA2 and GATA3 ensures an appropriate gene expression balance to promote self-renewal and expansion of TSPCs during early postimplantation mammalian development.

### In TSPCs GATA factors regulate key signaling components that mediate embryonic-extraembryonic signaling cross-talk

How does the loss of GATA2 and GATA3 in the extraembryonic trophoblast lineage impair development at an early postimplantation stage? Postimplantation embryonic development depends on BMP4 and Nodal signaling cross-talk between TSPCs and cells of the embryo proper (Beppu et al., 2000; Brennan et al., 2001; Kimura et al., 2000; Mishina et al., 1995; Rodriguez et al., 2005; Soares et al., 2005, 2008; Tam and Loebel, 2007; Winnier et al., 1995). TSPCs produce BMP4, which is required for primitive streak development (Murohashi et al., 2010; Streit et al., 1998). TSPCs also express the convertase enzymes PCSK3 (furin) and PCSK6, which process Nodal precursors to ensure proper embryo patterning (Guzman-Ayala et al., 2004). In addition to Nodal and BMP4, TSPCs also produce other factors that regulate the Wnt signaling pathway to control postimplantation development. For example, the secretory protein dickkopf 1 (DKK1), which negatively regulates the Wnt/β-catenin pathway, is required for gastrulation (Peng et al., 2008). Also, TSPCs express porcupine homolog (*Porcn*), which is necessary for the palmitoylation and secretion of functional Wnt molecules (Biechele et al., 2013).

RNA-seq analysis confirmed that along with *Bmp4*, the expression of additional GATA targets *Pcsk6* and *Dkk1* is repressed in *Gata*-DKO TSCs (Table S2), whereas the GATA target *Porcn* is expressed at very low levels in both control and *Gata-*DKO TSCs. As the loss of GATA factors strongly downregulated *Bmp4*, *Pcsk6* and *Dkk1* expression in TSCs, we tested whether GATA factor loss also impairs their expression in the primary TSPCs of a postimplantation embryo. Our gene expression analysis confirmed that loss of GATA factors strongly represses *Bmp4*, *Pcsk6* and *Dkk1* expression in TSPCs (Fig. 6E). By contrast, *Porcn* expression is induced in *Gata*-DKO TSPCs (Fig. 6E). These results indicate that GATA2 and GATA3 regulate the expression of BMP4, Nodal and Wnt signaling components in TSPCs, thereby facilitating embryonic-extraembryonic signaling cross-talk during early postimplantation development.

### GATA factors promote trophoblast differentiation by activating differentiation-specific genes

In mice, attachment of chorion to the allantois gives rise to GCM1^+^ (Basyuk et al., 1999) and DLX3^+^ (Morasso et al., 1999) labyrinth trophoblast progenitors (LTPs), which differentiate to syncytiotrophoblasts within the labyrinth zone (Fig. S1). In the EPC, ASCL2^+^, PRDM1^+^ and TPBPA^+^ progenitors (Mould et al., 2012; Simmons et al., 2007; Tanaka et al., 1997) arise. These progenitors subsequently differentiate into specialized trophoblast subtypes of the junctional zone, which contains TGCs, SpT cells and glycogen trophoblasts (GlyTs) (Fig. S1). As *Gata2* and *Gata3* deletion in E7.5 embryos leads to defective development of both the labyrinth and the junctional zones, we examined whether GATA factors exert a differentiation stage-specific function by promoting the transcription of key genes during trophoblast progenitor differentiation.

**Fig. 7.**
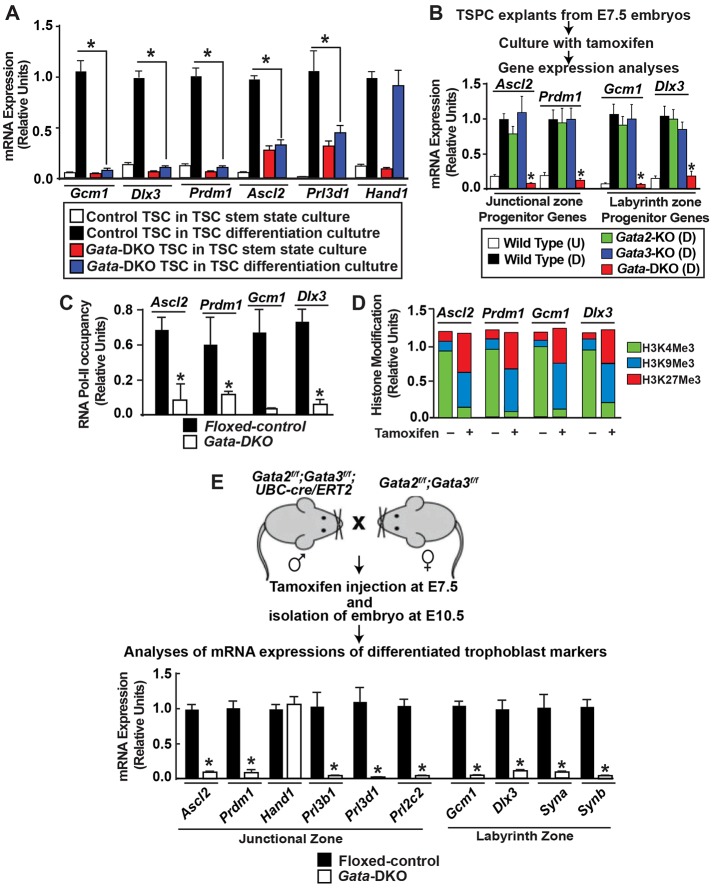
**Functional redundancy of GATA2 and GATA3 ensure proper gene expression during trophoblast progenitor differentiation.** (A) Control and *Gata*-DKO TSCs were cultured for 4 days in TSC differentiation culture conditions, and mRNA expression of trophoblast differentiation genes was determined by qRT-PCR. Mean±s.e., *n*=3, *P≤0.001. Except for *Hand1*, the induction of trophoblast differentiation markers was impaired in *Gata*-DKO TSCs. (B) Scheme of gene expression analyses in differentiating TSPCs. The plot shows impaired induction of key differentiation genes in *Gata*-DKO TSPCs. (C) RNA Pol II recruitment at promoters of key trophoblast genes in differentiating control and *Gata*-DKO TSPCs. Mean±s.e., *n*=3, *P≤0.01. (D) Maintenance of repressive histone markers (H3K9Me3 and H3K27Me3) and loss of H3K4Me3 at the promoter regions of key differentiation genes in differentiating *Gata*-DKO TSPCs. (E) Scheme of gene expression analyses in *Gata*-DKO placentae. The plot shows relative mRNA expression of trophoblast genes in control and *Gata*-DKO placentae. Mean±s.e., *n*=3 individual experiments, *P≤0.01.

The global transcriptome profile in TSCs revealed that GATA factors occupy chromatin domains of key trophoblast genes that are transcriptionally repressed in TSCs but are crucial to induce trophoblast lineage differentiation (Table S4). For example, we identified GATA factor occupancy at the chromatin domains of the GATA targets *Gcm1*, *Dlx3* and *Prdm1*, which are implicated in the development of syncytiotrophoblasts and TGCs (Basyuk et al., 1999; Berghorn et al., 2005; Hughes et al., 2004; Morasso et al., 1999; Mould et al., 2012). These genes are transcriptionally silent in TSCs (Table S4). However, their expression is induced in differentiated cultures of TSCs (Fig. 7A, Fig. S5D).

We examined whether *Gcm1*, *Dlx3*, *Prdm1* and other trophoblast differentiation markers are induced in *Gata*-DKO TSCs in FGF4-free differentiating culture conditions. In particular, we wanted to test whether genes such as *Ascl2*, *Prl3d1* and *Prl3b1*, which are expressed at higher levels in *Gata*-DKO TSCs in standard TSC culture conditions, are altered in expression in differentiating culture conditions. Intriguingly, these gene expression analyses revealed that differentiation potential is impaired in *Gata*-DKO TSCs (Fig. 7A, Fig. S5D). *Gcm1*, *Dlx3* and *Prdm1* remained suppressed in *Gata*-DKO TSCs when cultured in differentiating condition for several days (Fig. 7A, Fig. S5D). Also, mRNA expression of *Ascl2*, *Prl3d1* and *Prl3b1* was not further induced. However, *Hand1* (a GATA target), a gene that promotes TGC differentiation (Hemberger et al., 2004), was upregulated in *Gata*-DKO TSCs. Collectively, these results indicated that GATA2 and GATA3 promote trophoblast differentiation by directly regulating the expression of key differentiation genes.

To further validate the importance of GATA-mediated gene regulation during the differentiation of trophoblast progenitors to specialized trophoblast cells, we tested gene expression in *Gata*-DKO TSPC explants. For gene expression analysis in the differentiating TSPCs we isolated ExE/EPC explants from E7.5 embryos, cultured them in differentiating culture conditions and induced GATA gene deletion with tamoxifen (Fig. 7B). Gene expression analyses confirmed that the presence of either GATA2 or GATA3 is sufficient for the induction of key trophoblast differentiation genes, namely *Ascl2*, *Prdm1*, *Gcm1* and *Dlx*3 (Fig. 7B). However, the loss of both GATA2 and GATA3 impaired induction of these genes during the differentiation of TSPCs to specialized trophoblast cells (Fig. 7B). Furthermore, analysis of their chromatin domains in TSPCs revealed that loss of both GATA factors impaired RNA polymerase II (Pol II) recruitment and maintained repressive histone marks at these gene loci (Fig. 7C,D).

We also assessed gene expression in differentiated trophoblast cells of *Gata*-DKO placentae (Fig. 7E). Similar to the TSPC explant cultures, there was strong repression of *Gcm1*, *Prdm1*, *Dlx3* and *Ascl2* in *Gata-*DKO placentae (Fig. 7E). Loss of GATA factors strongly inhibited the expression of other TGC-specific genes including *Prl3b1*, *Prl3d1* and *Prl2c2* (Fig. 7E), an observation previously reported with individual GATA gene knockout placentae (Ma et al., 1997). Expression of syncytin A (*Syna*) and syncytin B (*Synb*), which are essential for labyrinth trophoblast syncytialization (Dupressoir et al., 2005, 2009, 2011), was also strongly repressed in *Gata*-DKO placentae. However, similar to *Gata*-DKO TSCs, expression of *Hand1* was not significantly altered in *Gata*-DKO placentae.

In summary, gene expression analysis in the TE, TSCs and primary trophoblast populations provided developmental snapshots of gene regulatory mechanisms of GATA2 and GATA3 during trophoblast lineage development. The loss-of-function analysis in *Gata*-DKO TSCs, TSPCs and placentae showed that the functional redundancy of GATA2 and GATA3 not only maintains trophoblast stem-state genes in TSPCs, but is also important to ensure the induction of key genes that initiate trophoblast progenitor differentiation to specialized trophoblast cells during placentation.

## DISCUSSION

Recently, multiple studies have implicated GATA2 and GATA3 in orchestrating gene expression patterns during trophoblast development (Bai et al., 2011; Home et al., 2009; Ma and Linzer, 2000; Ma et al., 1997; Ralston et al., 2010; Ray et al., 2009). However, owing to the lack of an overt phenotype in individual gene knockouts, the importance of trophoblast cell-specific GATA function during early mammalian development was difficult to interpret. Here, by studying a dual gene knockout model, we show that trophoblast-specific GATA2 and GATA3 functions are essential at multiple stages of early embryonic development. Our analyses also revealed that both GATA2 and GATA3 directly regulate a large number of trophoblast genes. These findings strongly support a complementary role of GATA2 and GATA3 during trophoblast development and explain the lack of an overt trophoblast phenotype in individual knockout studies.

Unlike *Gata2*-KO preimplantation embryos, *Gata3*-KO preimplantation embryos show a partial defect in blastocyst maturation, which is surprising as ChIP-seq analyses revealed that ∼90% of the GATA3 target genes in TSCs are also GATA2 targets. However, the ChIP-seq studies in TSCs provided snapshots of GATA factor binding at their chromatin targets in a large cell population. Thus, it is possible that during blastocyst maturation GATA3 and GATA2 exhibit dynamic chromatin occupancy, with more genes being bound by GATA3. Alternatively, a few genes that are selectively regulated by GATA3 might be more important for blastocyst maturation. Nevertheless, blastocyst formation in most of the *Gata2*-KO and the majority of *Gata3*-KO preimplantation embryos supports a functional redundancy of GATA2 and GATA3 during blastocyst maturation.

Developmental snapshots of gene expression in *Gata*-DKO TSCs and TSPCs showed that temporal fine-tuning of gene expression by GATA2/GATA3 regulates distinct stages of trophoblast development. For example, expression of *Prl3b1* and *Ascl2* is induced in *Gata*-DKO TE. By contrast, these genes are repressed in *Gata*-DKO differentiated trophoblast cells. These findings imply that GATA2 and GATA3 orchestrate trophoblast lineage development by ensuring developmental stage-specific gene expression patterns. How GATA factors fine-tune temporal gene expression in trophoblast cells is a subject of further study. One hypothesis is that, in response to different cellular signaling, GATA2/GATA3 form distinct protein-protein complexes at different chromatin domains, leading to alternative transcriptional outcomes. Also, pioneer transcription factors are known for RNA Pol II recruitment at both poised and transcribed genes (Hsu et al., 2015). Thus, it will be interesting to identify how GATA-dependent mechanisms regulate trophoblast chromatin at different stages of development and whether these mechanisms are conserved in multiple mammalian species, including human.

Interestingly, unlike labyrinth trophoblast and SpT cells, the development of TGCs, including parietal TGCs that separate the developing placenta from the maternal decidua, was not overtly affected in *Gata*-DKO placentae. Studies with mouse TSCs indicated that TGC development might be a default pathway, as withdrawal of FGF4 and other TSC self-renewal factors promotes the spontaneous differentiation of TSCs to TGCs (Tanaka et al., 1998). Also, expression of HAND1, a factor implicated in TGC development (Hemberger et al., 2004), is not dependent upon GATA factors. Thus, TGC development during mouse placentation is not absolutely dependent on GATA factor function. However, GATA2 and GATA3 are expressed in TGCs and regulate the expression of the TGC-specific genes *Prl2c2*, *Prl3d1* and *Prl3b1* (Ma et al., 1997). Thus, GATA factors might be important to maintain proper TGC functions that include the production of placental hormones and other secretory molecules to ensure the progression of pregnancy. Also, GATA2/GATA3 function might be important in the development of other TGC subtypes, including the invasive trophoblast population. Future studies with TGC-specific *Gata2*/*Gata3* deletion will provide more in-depth information regarding their importance in TGCs.

Another interesting finding is the complete lack of blood development in *Gata*-DKO placenta and embryo when gene deletion is induced at E7.5. The placenta is a major site of hematopoiesis and the placental hematopoiesis in mice begins after E9.0, when definitive multilineage progenitors appear (Alvarez-Silva et al., 2003). By contrast, mature hematopoietic stem cells in the embryo proper are first found at ∼E10.5 (Gekas et al., 2005; Ottersbach and Dzierzak, 2005). Although previous studies (Minegishi et al., 1998; Shi et al., 2014) reported that *Gata2* mRNA and protein are expressed in the lateral mesoderm of the ∼E7.5-8.0 mouse embryo, we found that at ∼E7.5 GATA2 and GATA3 proteins are mainly expressed in trophoblast cells (Fig. 1C). At this stage, no hematopoietic cell exists in the embryo proper or in the placenta. Also, *Gata*-DKO embryos under that experimental condition do not mature beyond E9.5, a developmental stage before the augmentation of definitive hematopoiesis in the embryo proper. Thus, it is possible that trophoblast cell-specific GATA function is required for proper hematopoiesis during embryonic development, a hypothesis that awaits future studies of GATA gene deletion in specific trophoblast cell types.

## MATERIALS AND METHODS

### Derivation of mouse TSC lines

*Gata2^f/f^;Gata3^f/f^;UBC-cre/ERT2/+* TSCs were established from E3.5 blastocysts according to the protocol of Tanaka et al. (1998) and cultured in the presence of FGF4 and heparin. *Gata2* and *Gata3* floxed alleles were efficiently excised from *Gata2^f/f^;Gata3^f/f^;UBC-cre/ERT2* TSCs by culturing the cells in the presence of tamoxifen (1 μg/ml). *Gata2^f/f^* and *Gata3^f/f^* TSCs were established in a similar fashion. Gene deletions were induced in these cell lines by transient transfections with Puro.Cre empty vector (Addgene plasmid 17408) (Kumar et al., 2008) according to a protocol described previously (Home et al., 2009). All cell lines were confirmed negative for contamination.

### Generation of conditional knockout mouse strains

All procedures were performed after obtaining IACUC approvals at the University of Kansas Medical Center. Female *Gata2^f/f^* mice (Charles et al., 2006) were mated with B6;129S-Tg(*UBC-cre/ERT2*)1Ejb/J male (JAX Lab, stock 007001) (Ruzankina et al., 2007) in order to generate *Gata2^f/+^;UBC-cre/ERT2*. In the next step, *Gata2^f/+^;UBC-cre/ERT2* female mice were bred with *Gata2^f/+^;UBC-cre/ERT2* males to generate *Gata2^f/f^;UBC-cre/ERT2*. Similarly, female *Gata3^f/f^* mice (Zhu et al., 2004) were used to generate *Gata3^f/f^;UBC-cre/ERT2*. Again, *Gata2^f/f^;UBC-cre/ERT2* and *Gata3^f/f^;UBC-cre/ERT2* mice were crossed to generate *Gata2^f/+^;Gata3^f/+^;UBC-cre/ERT2*. Subsequently, *Gata2^f/+^;Gata3^f/+^;UBC-cre/ERT2* males and females were crossed to generate the *Gata2^f/f^;Gata3^f/f^;UBC-cre/ERT2* strain.

### GATA gene deletion in postimplantation embryos

For gene deletion in postimplantation embryos, matings were set between *Gata2^f/f^;UBC-cre/ERT2* males and *Gata2^f/f^* females, *Gata3^f/f^;UBC-cre/ERT2* males and *Gata3^f/f^* females, and *Gata2^f/f^;Gata3^f/f^;UBC-cre/ERT2* males and *Gata2^f/f^;Gata3^f/f^* females. Once copulation plugs were confirmed (E0.5), intraperitoneal injections of 200 µl tamoxifen solution (10 mg/ml in corn oil) were administered on desired days for each of the females.

### Collection, culture and GATA gene deletion in preimplantation embryos

One-cell stage mouse embryos were harvested according to a published protocol (Home et al., 2012; Saha et al., 2013) and were cultured in KSOM (Millipore) in the presence or absence of 1 μg/ml tamoxifen at 37°C in a humidified chamber, maintained at 5% CO_2_ and 5% oxygen. For gene deletion using Cre recombinase-expressing vector, one-cell embryos were subjected to perivitelline space microinjection with lentiviral vectors expressing Cre recombinase. Further details are provided in the supplementary Materials and Methods.

### Explant culture of ExE/EPC

ExE/EPC regions were harvested from E7.5 pregnancies and cultured with or without FGF4 and heparin. Cultures were treated with tamoxifen to induce gene deletions. Outgrowths were allowed to grow for 72-96 h before RNA was prepared.

### TSC injections in embryos

*Gata*-DKO and control TSCs were transfected with pLKO.3G (Addgene plasmid 14748) and cell sorted (flow cytometry is described in the supplementary Materials and Methods) for strong GFP expression. Sorted cells were cultured in the presence or absence of tamoxifen. Gene deletions were confirmed by PCR. Eight to ten TSCs were microinjected into morulae or very early stage blastocysts using standard techniques. Embryos were allowed to grow to expanded blastocyst stage and micrographed for GFP fluorescence.

### First-trimester human placental tissue

De-identified and discarded first-trimester placental tissues were obtained from the Research Centre for Women's and Infants' Health (RCWIH) BioBank, Toronto, Canada after obtaining Institutional IRB approval from the University of Kansas Medical Center and Mount Sinai Hospital, Toronto, Canada.

### Genotyping, RT-PCR and immunofluorescence analyses

For genotyping, genomic DNA was prepared using tail tissue. Quantitative RT-PCR (qRT-PCR) analyses and immunofluorescence were performed following published protocols (Home et al., 2012; Saha et al., 2013). Further details are provided in the supplementary Materials and Methods. Oligonucleotides and antibodies are detailed in Tables S5 and S6.

### RNA-seq and ChIP-seq analyses

RNA-seq analyses were performed using the Illumina Genome Analyzer II platform with libraries that were prepared with purified mRNAs from control and *Gata*-DKO TSCs. Quantitative ChIP and ChIP-seq analyses were performed following published protocols (Home et al., 2009; Home et al., 2012). Further details are provided in the supplementary Materials and Methods.

### Statistical analyses

Independent data sets were analyzed by analysis of variance (ANOVA) using Student's *t*-test and are presented as mean±s.e.
